# Acquisition of *pcnB* [poly(A) polymerase I] genes via horizontal transfer from the β, γ-*Proteobacteria*

**DOI:** 10.1099/mgen.0.000508

**Published:** 2021-01-27

**Authors:** George H. Jones

**Affiliations:** ^1^​ Department of Biology, Emory University, Atlanta, GA 30322, USA

**Keywords:** horizontal gene transfer, *pcnB*, poly(A) polymerase, polyadenylation, tRNA nucleotidyltransferase

## Abstract

Poly(A) polymerases (PAPs) and tRNA nucleotidyltransferases belong to a superfamily of nucleotidyltransferases and modify RNA 3′-ends. The product of the *pcnB* gene, PAP I, has been characterized in a few β-, γ- and δ-* Proteobacteria *. Using the PAP I signature sequence, putative PAPs were identified in bacterial species from the α- and ε-* Proteobacteria* and from four other bacterial phyla (* Firmicutes *, *Actinobacteria *, *Bacteroidetes* and *Aquificae *). Phylogenetic analysis, alien index and G+C content calculations strongly suggest that the PAPs in the species identified in this study arose by horizontal gene transfer from the β- and γ-* Proteobacteria *.

## Data Summary

Protein IDs for all of the proteins utilized in this study are provided in Tables S1–S3 (available with the online version of this article) and in a previously published paper [1] (in Table S1). Protein sequences were obtained from the National Center for Biotechnology Information (NCBI) genome or protein databases (www.ncbi.nlm.nih.gov). The NCBI Gene Expression Omnibus database (www.ncbi.nlm.nih.gov/geo) was the source of the transcriptomic and proteomic data for the bacterial species studied. The ACLAME database (http://aclame.ulb.ac.be) was used to identify putative mobile genetic elements associated with horizontally transferred poly(A) polymerase genes.

**Impact Statement**

It has become increasingly apparent that the horizontal (or lateral) transfer of genetic information between species plays a significant role in cellular evolution and function. The present study demonstrates for what is believed to be the first time that the genes for bacterial polyadenylate polymerase [poly(A) polymerase I, PAP I] were acquired by horizontal transfer from the β- and γ-* Proteobacteria* to certain other bacterial phyla. Polyadenylation of RNA 3′-ends is an important step in the degradation of bacterial RNAs and the importance of this process is magnified by the evidence indicating that polyadenylation can regulate gene expression in bacteria. These degradative and regulatory functions may well extend to the newly identified species that have acquired PAP genes horizontally. The results presented here add to the existing data indicating that the polyadenylation of RNA 3′-ends catalysed by PAP I occurs widely in members of the domain Bacteria.

## Introduction

Polyadenylation of RNA 3′-ends, once thought to occur only in eukaryotes, is now known to play an important role in RNA metabolism in bacteria as well [2, 3]. In the β- and γ-* Proteobacteria *, the enzyme responsible for RNA 3′-polyadenylation is poly(A) polymerase I (PAP I), the product of the *pcnB* gene [4, 5]. Polyadenylation in bacteria is involved in the regulation of gene expression [6–8] and in RNA quality control [9–11] and 3′-tails facilitate degradation of RNAs by 3′−5′-exoribonucleases (reviewed by Mohanty and Kushner [12]). PAP I is a member of a nucleotidyltransferase superfamily (NTSF), which also includes the bacterial tRNA nucleotidyltransferases (TNTs) [13].

Polyadenylation of RNA 3′-ends has been shown to take place in bacterial species other than the β, γ-* Proteobacteria *. In the actinobacterium *Streptomyces coelicolor *, for example, 3′-tails were shown to be present on rRNAs and mRNAs [14]. Although *Streptomyces coelicolor* contains a protein with substantial sequence similarity to PAP I, biochemical assays demonstrated that this protein, SCO3896, is not a PAP, but rather is a TNT [15, 16]. The enzyme responsible for 3′-tail synthesis in *Streptomyces coelicolor* appears to be polynucleotide phosphorylase (PNPase) [15, 16]. PNPase has also been posited as the PAP in the *Cyanobacteria* [17] and in plant chloroplasts [18], and it is known that in mutants of *Escherichia coli* that lack PAP I, PNPase is the enzyme responsible for 3′-tail synthesis [19].

Recently, RNA 3′-polyadenylation has been shown to occur in the δ-proteobacterium, *Geobacter sulfurreducens *. A protein with substantial sequence similarity to PAP I was identified in the *Geobacter sulfurreducens* proteome and that protein was shown to possess PAP activity [20]. *Geobacter sulfurreducens* is also interesting in that it is a member of a group of bacterial species that contain separate CC- and A- adding TNTs as well as a PAP [1, 20].

RNA polyadenylation has also been demonstrated in the *Firmicute*, *Bacillus subtilis *. Like *Streptomyces coelicolor *, *Bacillus subtilis* contains a protein that bears sequence resemblance to PAP I. Again, like *Streptomyces coelicolor *, that protein is not a PAP but is a TNT [21]. Unlike *Streptomyces coelicolor *, a *Bacillus subtilis* mutant that lacks PNPase still adds 3′-poly(A) tails to RNAs [22]. Thus, there appears to be a different system for poly(A) tail synthesis in *Bacillus* as compared with the β, γ and δ-* Proteobacteria* and the *Actinobacteria *.

The foregoing observations raise the interesting question whether members of other bacterial phyla polyadenylate RNAs and, if so, what enzyme systems are used for that purpose. To approach an answer to this question, the proteomes of various bacterial phyla were examined via blast search using the PAP I signature sequence as the query. Those searches revealed PAP I-like proteins in a number of bacterial species, viz. the α- and ε-* Proteobacteria *, the *Actinobacteria *, *Firmicutes *, *Bacteroidetes* and *Aquificae *. Evidence is presented here that the *pcnB* genes in these species arose by horizontal gene transfer (HGT).

## Methods

### Protein sequences and phylogenetic analyses

Amino acid sequences of proteins of interest were retrieved from the National Center for Biotechnology Information (NCBI) genome or protein databases (www.ncbi.nlm.nih.gov). blast searches were conducted using the NCBI blast server with the blastp algorithm with default parameters.

Protein sequences were aligned using m-coffee with the default protein alignment parameters [23, 24]. The multiple sequence alignment (MSA) utilized to produce Fig. 2 is provided in clustal format as Fig. S1. Maximum-likelihood phylogenetic trees were reconstructed from the MSA using the proml program from phylip 3.695 [25]. The Jones–Taylor–Thornton substitution model [26] was used in proml for these analyses. The sequences were bootstrapped 1000 times and jumbled once. The tree output file from proml was entered into consense [25] to produce an unrooted consensus tree using M1 as the consensus type and 0.7 as the input fraction. Thus, the consensus tree shows only those nodes with bootstrap scores of at least 700. Hillis and Bull have argued that bootstrap values of ≥70 % generally correspond to a 95 % probability that the relevant clade is genuine [27]. The trees were rooted with TreeView 1.6.6 using the *Thermotoga maritima* TNT sequence as the outgroup. The *T. maritima* sequenced was trimmed to eliminate the Nrn and CBS domains, which are a part of the native protein sequence [1]. A total of 42 PAP I sequences and 21 TNT sequences were used in the construction of Fig. 2, and 17 PAP I sequences and 103 TNT sequences were used in constructing Figs 5 and S4 [see Table S1 and a previously published paper [1] (Table S1) for lists of species whose sequences were used to construct the figures]. TNT sequences for six *Bacteroidetes* species (Table S3) were included with the others used for the production of Figs 5 and S4.

In addition to the maximum-likelihood phylogenies produced from the TNT and PAP sequences, a maximum-likelihood tree was also generated from the PNPase protein sequences obtained for the species listed in Tables S1 and S3. The protein IDs for these sequences are listed in Table S2. The tree was reconstructed with phylip as described above and rooted with the PNPase sequence from *T. maritima *. Protein sequences as text files and alignments in clustal or phylip formats for all of the analyses described herein are available from the author on request.

### Alien index and G+C content calculations

Alien indices, indicative of the likelihood of horizontal transfer of *pcnB* genes from the donor to the recipient species listed in Table 1, were calculated as described previously [1, 28]. Details of the calculations are provided in Results and Discussion. G+C contents of relevant genomes were obtained from the NCBI genome database (www.ncbi.nlm.nih.gov), and G+C contents of relevant genes and other sequences were calculated using the ENDMEMO DNA/RNA GC Content Calculator (http://www.endmemo.com/bio/gc.php).

**Table 1.** Alien indices for the putative PAPs identified in this study The method used for calculating the alien indices is described in the text. Greek letters in parentheses after the species names in the first, second and fourth columns indicate the proteobacterial class to which that species is assigned. Letters in parentheses after the protein IDs in the second column are the species abbreviations used in the MSA and in Figs 2 and 3. All of the proteins from species listed in the second column are annotated as PAPs in the NCBI protein database, whereas the proteins from the species listed in the fourth column are, with one exception, annotated as TNTs.

NPPC species	Potential donor	E value (% identity) (% coverage)	Potential recipient	E value (% identity) (% coverage)	Alien index
* Campylobacter jejuni * NCTC12850 (ε)	* Rahnella* sp. JUb53 (γ) PAP (WP_132964877.1) (Ras)	0 (98.4) (100)	* Campylobacter jejuni* 119462 (ε) TNT (ECR3422709.1)	1e−26 (34.1) (31)	400
* Mesorhizobium* sp. (α) isolate N.Ca.ET.004.03.1	* Enterobacter cloacae* (γ) PAP (WP_072057724.1) (Ecl)	0 (99.6) (100)	* Mesorhizobium ciceri* (α) TNT (WP_0271039413.1)	5e−20 (31.3) (50)	413
* Rhodobacteraceae* bacterium CH30 (α)	* Neisseriaceae* bacterium B2N2-7 (β) PAP (WP_160795087.1) (Nba)	0 (99.6) (100)	* Rhodobacteraceae* bacterium 63 075 (α) TNT (WP_117150976.1)	2e−27 (35.4) (51)	399
* Pedobacter himalayensis* HHS22 (Bacteroidetes)	* Enterobacter cloacae* (γ) PAP (OOK65942.1) (Ecl)	0 (100) (100)	* Bacteroidetes* bacterium (* Bacteroidetes *) HD-domain containing protein (TAL69253.1)	1e−25 (31.3) (51)	403
* Streptococcus pneumoniae * NCTC7978 (*Firmicute*)	* Escherichia coli* (γ) PAP (KZJ88923.1)	0 (99.8) (100)	* Streptococcus* sp*.* 263_SSPC (*Firmicute*) TNT (WP_048782664.1)	1e−36 (37.6) (54)	378
* Streptococcus dysgalactiae* subsp* . equisimilis * NCTC11565 (*Firmicute*)	* Pseudomonas aeruginosa* (γ) PAP (WP_033998106.1)	0 (100) (100)	* Streptococcus danieliae* (*Firmicute*) TNT (WP_160332659.1)	3e−34 (36.5) (51)	383
* Listeria monocytogenes* str. 104 657 (*Firmicute*)	* Escherichia coli* (γ) PAP (KZJ88923.1)	0 (99.6) (100)	* Listeria monocytogenes * (*Firmicute*) TNT (EAC7660737.1)	3e−29 (29.8) (51)	395
*'Empedobacter haloabium*' (* Bacteroidetes *)	* Lautropia* sp. SCN 69-89 (β) PAP (ODS98441.1) (Las)	0 (91.3) (99)	* Empedobacter brevis * (* Bacteroidetes *) TNT (VDH16430.1)	9e−19 (32.3) (44)	419
* Helicobacter pametensis * NCTC12888 (ε)	* Eikenella corrodens* (β) PAP (WP_049259245.1) (Eik)	0 (96.7) (100)	* Helicobacter ailurogastricus* (ε) TNT (WP_053945361.1)	7e−35 (35.0) (57)	382
* Mumia flava * MUSC201 (* Actinobacteria *)	* Ralstonia pickettii* (β) PAP (MRS99148.1) (Rpi)	0 (98.3) (100)	* Cellulomonas* sp*.* HZM (* Actinobacteria *) TNT (WP_081861460.1)	5e−28 (35.5) (48)	398
* Mycobacterium abscessus* subsp* . abscessus* str. 226 (* Actinobacteria *)	* Bordetella bronchiseptica* str. NCTC8762 (β) PAP (WP_015065010.1) (Bbr)	0 (100) (100)	* Rhodococcus qingshengii* str. S-E5 (* Actinobacteria *) TNT (WP_133367189.1)	5e−28 (32.9) (55)	398
* Mycobacterium tuberculosis* str. 2926STDY5723586 (* Actinobacteria *)	* Morganella morganii* (γ) PAP (WP_073970191.1) (Mmo)	0 (99.8) (100)	* Rhodococcus qingshengii* str. S-E5 (* Actinobacteria *) TNT (WP_133367189.1)	6e−27 (32.5) (51)	400
* Streptomyces cavourensis * YBQ59 (* Actinobacteria *)	* Achromobacter* sp. DH1f (β) PAP (WP_025136726.1) (Acb)	0 (100) (100)	* Streptomyces* sp. WAC00263 (* Actinobacteria *) TNT (OMP24185.1)	4e−28 (33.0) (48)	397
* Chryseobacterium* sp. 18 061 (* Bacteroidetes *)	* Citrobacter* sp. 18 056 (γ) PAP (WP_159771621.1) (Cis)	0 (99.4) (100)	* Chryseobacterium* sp. F5649 (* Bacteroidetes *) HD-domain containing protein (WP_124801980.1)	2e−18 (29.9) (48)	420
* Aquificaceae* bacterium isolate MAG 28 Ga0226836_10001573 (* Aquificae *)	* Leucothrix mucor* (γ) PAP (HFC91403.1) (Lmu)	0 (82.4) (97)	* Aquificae* bacterium (* Aquificae *) TNT (RLD95637.1)	1e−48 (50.1) (45)	350

### Search of DNA sequences for putative mobile genetic elements (MGEs)

The ACLAME software (http://aclame.ulb.ac.be/) [29, 30] was used to search for potential MGEs in the vicinity of the *pcnB* genes identified in this study. Genomic sequences were obtained from the NCBI genome database, and regions of 5 kb flanking the *pcnB* genes upstream and downstream were searched for putative MGEs using the ACLAME blast feature.

## Results and Discussion

### β, γ-PAP I signature sequences identify putative PAPs in other bacterial taxa

PAPs and TNTs both function as RNA 3′-nucleotidyltransferases [13]. Some years ago, Martin and Keller identified amino acid sequences that distinguish the two types of NTSF [31]. In particular, Martin and Keller described a signature sequence that is diagnostic of bacterial PAPs. The consensus PAP I signature sequence is [LIV][LIV]G[RK][RK]Fx-[LIV]h[HQL][LIV], where x is any amino acid and h is a hydrophobic residue. The crystal structure of *E. coli* PAP I has been solved by the Tomita group and it was shown that the signature sequence is located in the β-turn of the catalytic domain of the enzyme [32]. When the two Arg residues that are contained in the *E. coli* PAP signature sequence were changed to Ala residues, the AMP incorporating activities of the resulting proteins were reduced to 10–30 % of wild-type levels. The authors concluded that the Arg residues are involved in RNA binding and in the catalysis of AMP incorporation [32].

**Fig. 1.**
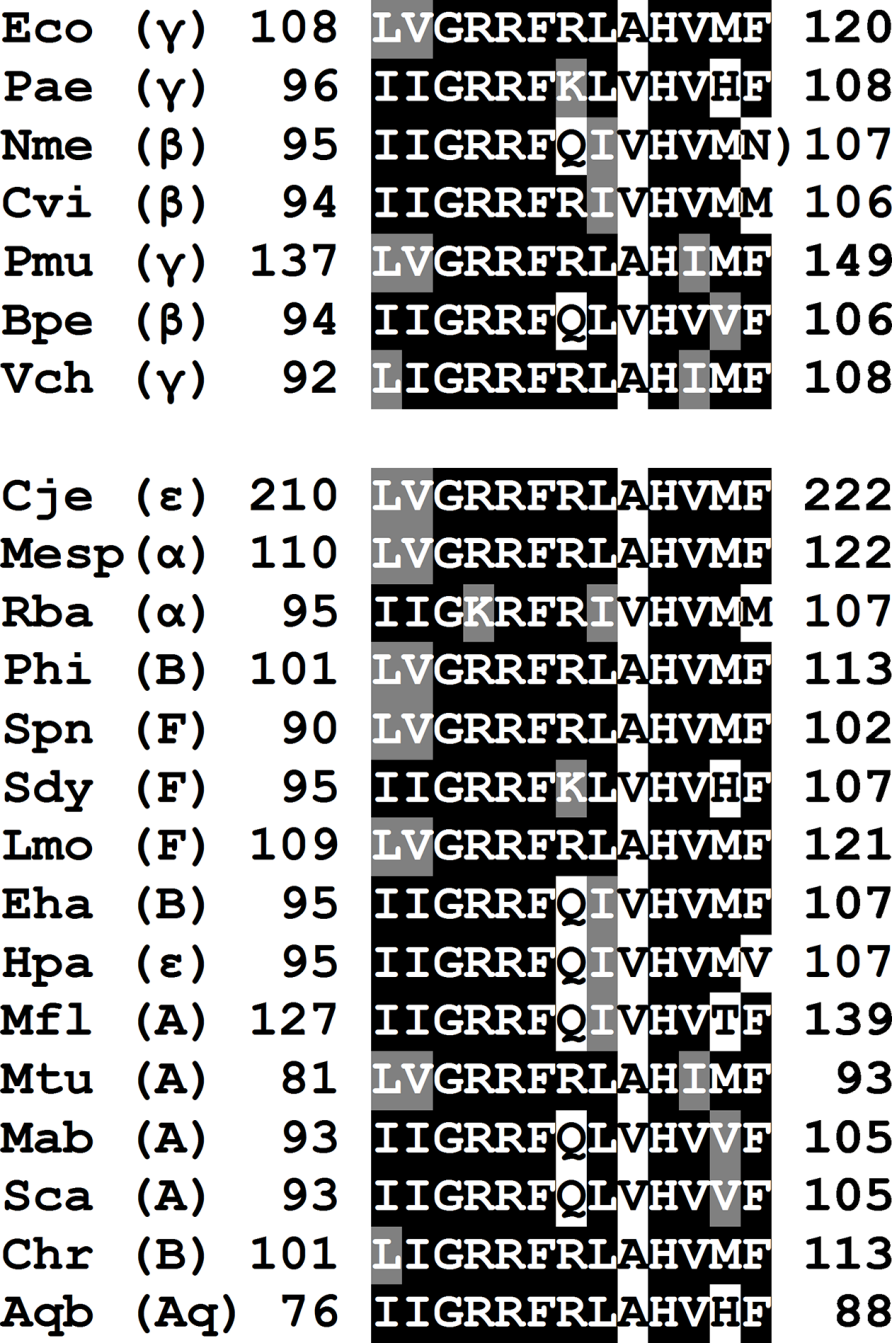
PAP I signature sequences. The sequences in the first group, representing the β, γ-proteobacterial PAPs, were used as blast queries to identify proteins from other bacterial classes and phyla. Those classes and phyla are specified using the following abbreviations: ε, ε-* Proteobacteria *; α, α-* Proteobacteria *; B, *Bacteroidetes *; F, *Firmicutes *; A, *Actinobacteria *; Aq, *Aquificae *.

The signature sequences for several β- and γ-proteobacterial PAPs were used as queries in blast searches of the proteomes of other bacterial taxa. The query sequences are shown in the upper portion of Fig. 1 and are derived from *E. coli *, *Pseudomonas aeruginosa *, *Neisseria meningitidis *, *Chromobacterium violaceum *, *Pasteurella multocida, Bordetella pertussis* and *Vibrio cholerae *. The results of these searches are presented in the lower portion of Fig. 1. The blast analysis identified proteins from 15 species with significant sequence similarity (80–100 % identity over the entire amino acid sequence) to the β, γ-proteobacterial PAPs. Those species are listed in Table S1, and represent the α- and ε-* Proteobacteria* and the bacterial phyla *Bacteroidetes *, *Firmicutes *, *Actinobacteria* and *Aquificae *.

It should be noted that all of the proteins whose signature sequences are shown in the lower portion of Fig. 1 are annotated as PAPs in the NCBI databases, and that Martin and Keller identified a number of taxa, in addition to the β, γ-* Proteobacteria *, whose species contained proteins bearing the PAP signature sequence [31]. However, Martin and Keller proposed a different mechanism for the evolution of those proteins than the one presented below. It should also be noted that only the activities of *E. coli* PAP I [5] and the corresponding enzyme from *Geobacter sulfurreducens* [20] have been verified experimentally. Genetic evidence for the role of *pcnB* in RNA polyadenylation in *Pseudomonas fluorescens* has been presented [33].

### Phylogenetic analysis of the putative PAPs

As a first step toward understanding the evolution of PAPs in the species identified in the blast searches, a phylogenetic analysis was performed. The sequences were first aligned with m-coffee and that MSA (Fig. S1) was then used as an input for the phylip software.

**Fig. 2.**
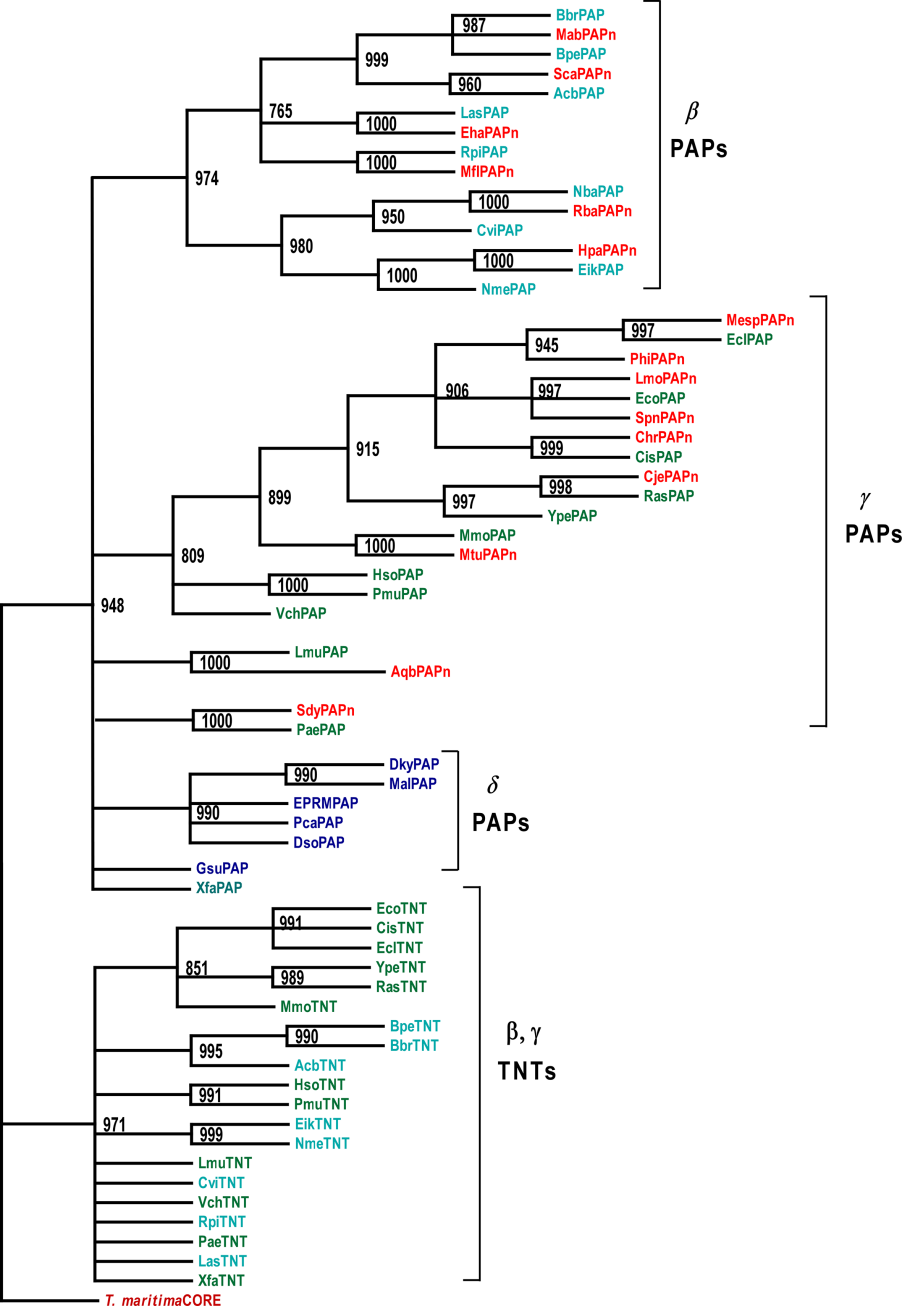
Maximum-likelihood phylogenetic tree relating the PAPs and TNTs referred to in the text. The tree was reconstructed using phylip 3.695 as described in Methods and rooted using the *T. maritima* core TNT sequence as the outgroup. Bootstrap scores are shown at the nodes. The putative PAPs identified in the present study are indicated in red (NPPCs, PAPs identified in the species listed in Table S1, from phyla other than the β,γ-* Proteobacteria *), cyan (PAPs from the β-* Proteobacteria* indicated as donor species in Table 1) and green (PAPs from the γ-* Proteobacteria* indicated as donor species in Table 1). PAP I sequences from the δ-* Proteobacteria* are shown in blue. Sequences of selected β- and γ-proteobacterial TNTs were included in the phylogenetic analysis for comparison. Those from the β-* Proteobacteria* are shown in cyan and those from the γ-* Proteobacteria *, in green. Note that only the activities of the *E. coli* and *Geobacter sulfurreducens* PAPs have been verified experimentally. See text for additional details.

The maximum-likelihood phylogenetic tree produced is shown in Fig. 2. The species whose PAPs were identified by the blast searches (referred to as a group below as NPPC species, for New Phyla PAP-Containing species) are indicated in red in Fig. 2. Note that the MSA also contained PAP sequences other than those shown in the lower portion of Fig. 1. Those sequences, from the β- and γ-* Proteobacteria *, are represented by the species abbreviations indicated in cyan and green, respectively, in Fig. 2, and their derivation will be described in greater detail below. There is evidence that some δ-* Proteobacteria* contain PAPs [1, 20] and those species, identified in a previous study [1], are indicated in blue in Fig. 2. Altogether, 42 different putative PAP sequences were used to generate the figure.

The tree was rooted with the ‘core’ CCA-adding sequence of the *T. maritima* CCA-adding enzyme. The core sequence lacks the Nrn and CBS domains that are included in the native amino acid sequence [1], and it has been shown that those accessory domains are not required for the CCA-adding function of the protein [34]. The *T. maritima* enzyme was used as the outgroup to root the tree because this bacterial species has been shown to be at or near the base of bacterial phylogenetic trees based on small subunit ribosomal RNAs (www.arb-silva.de/projects/living-tree and [35]), conserved signature indels [36], ribosomal protein, elongation factor and RNA polymerase subunit sequences [37], and core genome sequences [38]. The *T. maritima* sequence was used as the outgroup in a previous phylogenetic analysis of bacterial NTSFs [1]. For comparison purposes, a number of β- and γ-proteobacterial TNT sequences were also included in the phylogenetic analysis. These are also shown cyan and green, respectively, in Fig. 2.

It is apparent that the putative PAPs are found in three clusters in the maximum-likelihood phylogenetic tree, representing the β-, γ- and δ-* Proteobacteria *. This observation indicates that the putative PAPs from the NPPCs (the α- and ε-* Proteobacteria *, *Bacteroidetes *, *Firmicutes *, *Aquificae* and *Actinobacteria *) have a strong phylogenetic relationship to the proteobacterial PAPs (see further below).

**Fig. 3.**
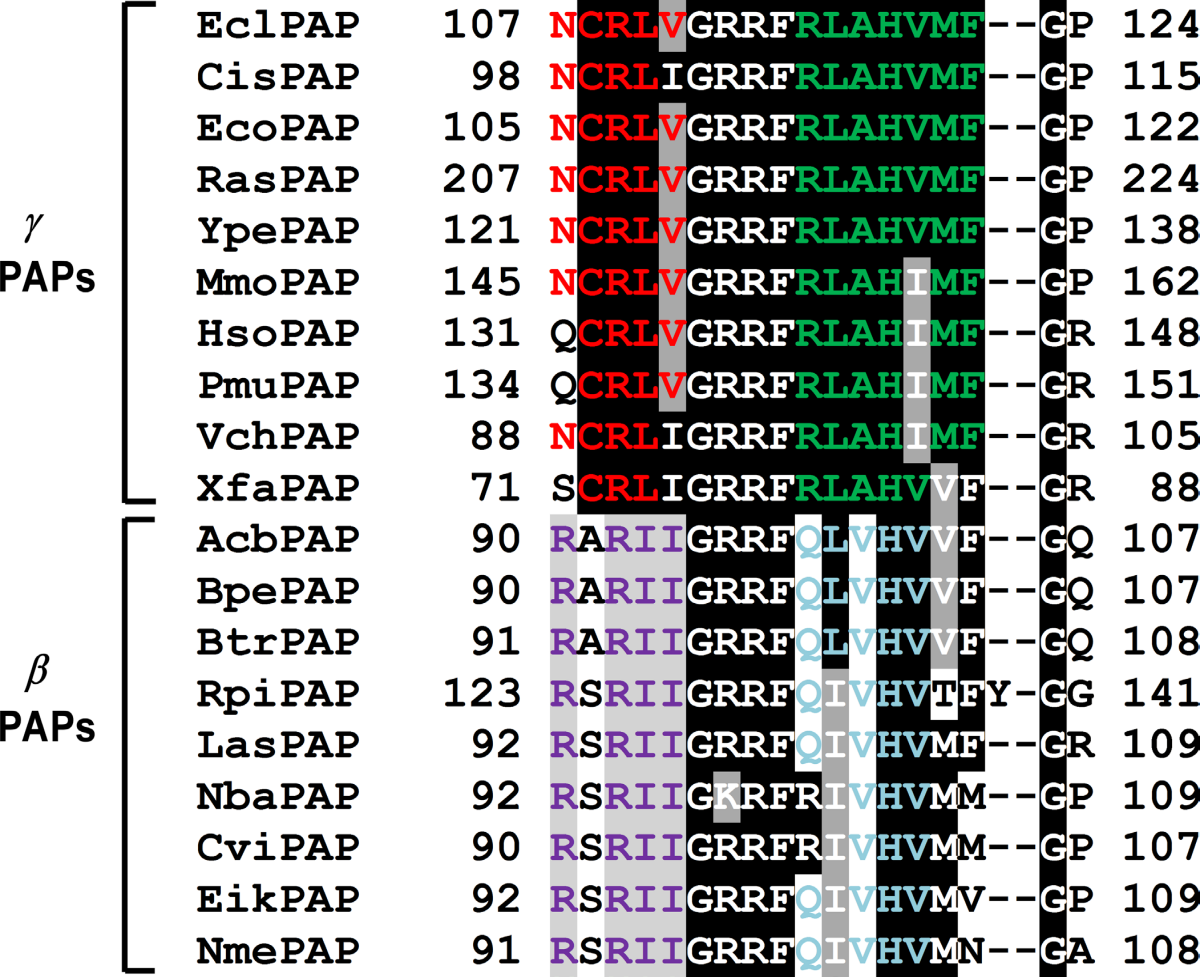
Sequence regions from the MSA of the bacterial PAP I signature sequences analysed in Fig. 2. The sequences shown are situated in the head region of PAP I, based on the crystallographic structure obtained for *E. coli* PAP I [32]. Colours indicate highly conserved amino acid residues in the γ- or β-proteobacterial PAPs that are represented in the figure.

Fig. 2 is also noteworthy in that it is possible to assign the β- and γ-proteobacterial PAPs to separate clades. To understand further the basis for this observation, the signature sequences of selected β- and γ-PAPs were grouped and aligned. That alignment is shown in Fig. 3. It is apparent that there are conserved sequences that are present in the β- and γ-PAPs that distinguish the two classes from each other, at least for the species whose sequences are shown in Fig. 3. For example, the RLAH sequence is completely conserved in all the γ-PAPs that are shown while the corresponding sequence differs and shows somewhat less conservation in the indicated β-PAPs.

### Evidence for the horizontal transfer of *pcnB* (PAP I) genes from the β, γ-* Proteobacteria* to other bacterial taxa

Available evidence suggests that PAPs evolved in the β- and γ-* Proteobacteria* from an ancestor of the CCA-adding TNT found in those organisms [1, 31, 32]. PAPs appear to be ubiquitous in the β- and γ-* Proteobacteria *; for example, over 70 species of bacteria from these two classes were examined in the present study and in every case those species contained both a TNT and a PAP. This is not the case in the δ-* Proteobacteria *, in which some species appear to contain only a CCA-adding TNT, some contain separate CC- and A-adding TNTs but no PAP, and some contain separate CC- and A-adding TNTs and a PAP [1].

Moreover, there is biochemical evidence suggesting that bacterial PAPs evolved from TNTs. Cho *et al*. demonstrated that point mutations in three residues situated in the neck region of the *Geobacillus stearothermophilus* TNT modified the activity of the enzyme. When R194, M197 and E198 were all replaced by alanines, the resulting mutant protein acquired the ability to add poly(A) tails to a tRNA substrate [39].

In addition, in domain swapping experiments, Betat *et al*. identified a region of 27 amino acids that was responsible for conferring CCA- or poly(A)-adding activity on the resulting chimaeras [40]. When the N-terminal region of *E. coli* PAP I, lacking those 27 amino acids, was fused to the C-terminal region of the *E. coli* TNT, the resulting chimaera functioned as a CCA-adding enzyme. In contrast, when those 27 amino acids were included in the N-terminal domain of the PAP, the fusion protein functioned as a PAP [40]. The 27 amino acids in question reside in the body region of the PAP structure (see Fig. S2). Toh *et al*. argue that those 27 amino acids, along with those found in adjacent α-helices in PAP I, interact to determine nucleotide specificity [32].

Taken together, the foregoing analyses argue for the vertical transmission of bacterial *cca* (TNT) genes. A different evolutionary scheme has been presented for the TNTs and PAPs from the δ-* Proteobacteria *. The CC- and A-adding enzymes from those species appear most closely related to the corresponding enzymes from species that are near the base of the ribosomal RNA-based phylogenetic tree, e. g. the *Aquificae* [1]. The phylogenetic incongruence observed for these proteins suggested that they arose by horizontal rather than vertical inheritance [1]. Similarly, the PAPs from the δ-* Proteobacteria* appeared in clades containing PAPs from the β, γ-* Proteobacteria* (cf. Fig. 2 and [1]). These and other observations suggested that like the CC- and A-adding enzymes, the PAPs in the δ-* Proteobacteria* arose by horizontal rather than vertical inheritance [1].

The question that arises from consideration of the data provided in Figs 1 and 2 is whether the putative PAPs identified in the present study also arose by HGT. To obtain additional insight into this question, and as was done in the previous study of the bacterial NTSFs [1], alien indices [28] were calculated for the 15 putative PAPs identified here.

The alien index was originally defined by Gladyshev *et al*. [41] and the principle has been refined by Rancurel *et al*. [28]. The latter authors defined the alien index as :

AI = ln(best recipient E value+1E^−200^) – ln(best donor E value+1E^−200^)

The E values are determined from blast searches using appropriate query sequences and donor and recipient sequences. In the case of the bacterial PAPs, the query sequence used was that of the PAP whose gene was suspected of acquisition via HGT, viz. the 15 NPPC PAP sequences. The donor species would be the ‘alien’ species, the potential source of the horizontally transferred gene. The recipient species would be one related to the query species, into which the ‘alien’ gene was transferred during evolution.

Gladyshev *et al*. proposed an alien index value of ≥45 as a strong indicator of HGT. Using a different and larger dataset, Rancurel *et al*. proposed three categories for classifying blast results used in the calculation of the alien index: (i) very likely HGT (alien index >30 and <70 % identity to candidate donor); (ii) possible HGT (alien index >0 and <70 % identity to candidate donor); and (iii) likely contamination (alien index >0 and ≥70 % identity to candidate donor). The stipulation that the query sequence should be <70 % identical to the candidate donor sequence only applies to HGT from prokaryotes to eukaryotes and should not affect the analyses presented here [28, 42].

Table 1 presents the results of the alien index calculations for the species indicated in Fig. 1 and Table S1. First, it is apparent from the table that in every case, the most likely potential source of the horizontally transferred *pcnB* gene was a β- or γ-proteobacterium. These are the species indicated in cyan and green in Fig. 2, and it is clear from the figure that these species appear in each case in the clade that contains the PAP identified in the blast search. It is especially noteworthy that for all 15 species listed in Table 1, the E value for the potential donor PAP was 0, and the query and potential donor PAPs were 80–100 % identical over their entire sequences.

Returning to the phylogenetic analysis, Koski and Golding pointed out some years ago that the closest hit from a blast search might not produce the nearest neighbour in a phylogenetic analysis [43]. These authors noted the importance of the congruence between sequence relatedness as determined by blast searches and phylogenetic proximity. It is important to note, therefore, that for all 15 proteins obtained for the species listed in Table 1, the closest neighbour in the maximum-likelihood tree of Fig. 2 is, indeed, the PAP from the donor species identified by the blast search. The potential recipient in each case presented in Table 1 was a related species that did not contain a PAP. The most closely related sequence in each case was the TNT that was present in the potential recipient.

Using the E values obtained for the potential and donor proteins, alien indices were calculated as described above and are shown in the last column of Table 1. The maximum value possible for the alien index is 460.5. It can be seen that the alien indices calculated for the species identified in the present study ranged from 350 to 420, a strong indication that the *pcnB* genes in the species identified in the blast searches described herein were acquired by HGT from species of β, γ- *Proteobacteria *.

As a negative control, the alien index values were calculated for PNPase from the NPPC species. PNPase is widely distributed among the bacteria and there is no evidence that *pnp* genes were inherited by HGT [44]. When, for example, the *Campylobacter jejuni* NCTC12850 PNPase was used as a blast query with *Rahnella* sp. JUb53 as the subject, an E value of 6e−161 was obtained for the comparison with the *Rahnella* PNPase. When the blast analysis was performed with *Campylobacter jejuni* strain 119462 (the potential recipient in the horizontal transfer of the *Rahnella pcnB* gene; Table 1) as the subject an E value of 0 was observed, indicating near identity of the PNPases from *Campylobacter jejuni* NCTC12850 and strain 119462. The alien index value calculated from these results is −91.6, a value which argues strongly against the acquisition of the *Campylobacter jejuni* NCTC12850 *pnp* gene from *Rahnella* Jub53 by HGT.

A phylogenetic analysis was performed as described in Methods, with the PNPase sequences from the species used to construct Fig. 2. The maximum-likelihood tree resulting from that analysis is depicted in Fig. S3. It is apparent that no phylogenetic incongruity was observed for the PNPases used in this study. All of the PNPases cluster in the clades corresponding to the phylum from which the sequences were obtained. This is in clear contrast to the results for the PAP I proteins from the NPPC species, which derive from members of the β- and γ-* Proteobacteria* (Fig. 2, Table 1). These results support the use of the PNPases as a negative control in the alien index calculations.

An anomalous (i.e. significantly different from genome average) G+C-content for particular genes is frequently considered diagnostic of HGT events [45–47]. To provide additional data supporting the potential HGT of the *pcnB* genes from the β- and γ-* Proteobacteria* to the NPPC species, G+C contents were determined for the genomes of those species and those of the putative donors, as well as for the *pcnB* and *pnp* genes of the NPPC and donor species. As mentioned above, there is no evidence to suggest that *pnp* has been inherited horizontally, so its G+C composition would be expected to match that of the species genome.

Table 2 shows the results of the G+C content analysis. It is apparent that for each of the 15 *pcnB* genes identified in this study, the G+C content is closer to that of the corresponding gene from the putative donor species than to the genome or the *pnp* gene from the NPPC species. The difference in several cases is especially dramatic. For example, the genome and *pnp* genes from '*Empedobacter haloabium*' have approximately 35 % G+C content, whereas the *pcnB* gene in that organism has 73 % G+C, identical to the G+C content of the *pcnB* gene from the putative donor species, *Lautropia* sp. SCN 69-89.

**Table 2.** G+C content (mol%) of genomes, *pnp* and *pcnB* genes for species posited to have acquired *pcnB* genes by horizontal transfer

NPPC species	NPPC genome	*pnp*	*pcnB*	Donor species	Donor genome	*pnp*	*pcnB*
* Campylobacter jejuni * NCTC12850	30.4	36.3	**56.7**	* Rahnella* sp. JUb53	52.2	52.4	**56.3**
* Mesorhizobium* sp. isolate N.Ca.ET.004.03.	60.5	63.0	**57.5**	* Enterobacter cloacae *	55.0	55.0	**57.5**
* Rhodobacteracea *e bacterium CH30	58.9	57.2	**63.6**	* Neisseriaceae* bacterium B2N2-7	59.3	56.8	**61.1**
* Pedobacter himalayensis* HHS22	42.1	39.9	**57.4**	* Enterobacter cloacae *	55.0	55.0	**57.5**
* Streptococcus pneumoniae * NCTC7978	39.6	44.5	**56.0**	* Escherichia coli *	50.6	54.1	**56.3**
* Streptococcus dysgalactiae* subsp* . equisimilis* NCTC11565	39.4	44.3	**67.9**	* Pseudomonas aeruginosa *	66.2	64.0	**67.7**
* Listeria monocytogenes* str. 104657	37.9	39.7	**55.7**	* Escherichia coli *	50.6	54.1	**56.3**
*'Empedobacter haloabium*'	32.7	36.5	**71.3**	* Lautropia* sp*.* SCN 69-89	65.5	65.6	**71.3**
* Helicobacter pametensis * NCTC12888	40.1	42.9	**63.3**	* Eikenella corrodens *	55.7	59.6	**63.7**
* Mumia flava * MUSC201	72.0	69.9	**66.9**	* Ralstonia pickettii *	63.8	62.7	**67.8**
* Mycobacterium tuberculosis * 2926STDY5723586	65.6	65.5	**56.0**	* Morganella morganii *	51.0	53.1	**55.9**
* Mycobacterium abscessus* subsp* . abscessus* str. 226	64.1	38.2	**67.5**	* Bordetella bronchiseptica* str. NCTC8762	68.2	66.5	**67.5**
* Streptomyces cavourensis * YBQ59	72.1	67.9	**67.0**	* Achromobacter* sp. DH1f	65.8	66.8	**68.5**
* Chryseobacterium* sp. 18061	38.0	40.9	**57.2**	* Citrobacter* sp. 18056	51.9	55.9	**57.4**
* Aquificacea *e bacterium isolate MAG 28 Ga0226836_10001573	49.4	53.0	**40.9**	* Leucothrix mucor *	43.7	48.3	**43.1**

### MGEs located within 5 kb of the *pcnB* genes in the NPPC species

MGEs are the agents that facilitate HGT [48, 49]. It was of interest, therefore, to determine whether DNA sequences representing putative MGEs were situated in the vicinity of the *pcnB* genes identified in the NPPC species in this study. To this end, genomic sequences were scanned, 5 kb upstream and downstream of the *pcnB* genes, using the ACLAME database and software [29, 30]. This database contains a searchable collection of MGEs of various types (plasmids, prophages, viruses, transposons and other elements).

Results of the ACLAME blast analyses are shown in Table 3. It is apparent that putative MGEs were found within 5 kb upstream and/or downstream of the *pcnB* genes in 11 of the 15 NPPC species. The E values for these MGEs ranged from 1e–03 to 2e–40, and their lengths ranged from approximately 20 to over 200 bp. Putative plasmid, prophage and viral sequences were observed.

**Table 3.** Putative MGEs situated within 5 kb of the *pcnB* genes in the NPPC species Sequences were searched using the blast feature of the ACLAME server. Putative MGEs are identified using the ID number for each element indicated in ACLAME. The distance from *pcnB* was measured from the 3′-base in the *pcnB* stop codon to the 5′-base in the MGE sequence or from the 5′ -base in the *pcnB* start codon to the 3′-base in the MGE sequence. For some species, only the putative MGEs with the lowest E values are shown. Others with higher E values were found, but for convenience were omitted from the table. The putative hosts for each element are listed as provided in ACLAME. Numbers in parentheses in columns 1 and 3 indicate the G+C contents of the relevant genome and the MGEs found in that species, respectively. nd, None detected.

NPPC species and G+C content (mol%)	Element ID	Length (bp) and G+C content (mol%)	Distance from *pcnB* (bp)	Host	E value (% identity)
* Campylobacter jejuni* NCTC12850 (**30.4**)	Plasmid 21669 Plasmid 15088	27 (**74.1**) 22 (**86.4**)	2301 (stop) 4469 (start)	* Ralstonia solanacearum* (β) * Deinococcus radiodurans* R1	1e−03 (96) 3e−03 (100)
* Mesorhizobium* sp. isolate N.Ca.ET.004.03.1 (**60.5**)	Plasmid 16453	22 (**59.1**)	1896 (stop)	* Halobacterium* sp. NRC-1	4e−03 (100)
*Rhodobacteriaceae* bacterium CH30 (**58.9**)	Plasmid 21162 Plasmid 22212 Plasmid 133739	22 (**86.4**) 22 (**68.2**) 33 (**72.7**)	81 (stop) 4101 (start) 4681 (start)	* Ralstonia solanacearum* (β) * Ralstonia solanacearum* (β) * Sphingomonas* sp. KA1 (α)	3e−03 (100) 7e−03 (34) 9e−03 (90)
* Pedobacter himalayensis* HHS22 (**42.1**)	Prophage 172094	27 (**63.0**)	642 (start)	* Desulfovibrio desulfuricans* subsp. *desulfuricans* str. G20	2e−03 (96)
* Streptococcus pneumoniae* NCTC7978	nd	–	–	–	–
* Streptococcus dysgalactiae* subsp* . equisimilis * NCTC11565 (**39.4**)	Prophage 168468 Plasmid 142336 Plasmid 142359 Plasmid 125959 Plasmid 21137	52 (**61.5**) 46 (**65.2**) 41 (**65.9**) 75 (**62.7**) 77 (**71.4**)	4494 (stop) 4489 (stop) 5032 (stop) 1739 (stop) 1536 (start)	* Burkholderia vietnamiensis* G4 * Rhodococcus* sp. RHA * Rhodococcus* sp. RHA * Rhizobium etli* CFN 42 * Ralstonia solanacearum* GMI1000	2e−06 (88) 3e−05 (89) 1e−04 (90) 7e−06 (84) 9e−12 (87)
* Listeria monocytogenes *	nd	–	–	–	–
*'Empedobacter haloabium*' (**32.7**)	Plasmid 11576 Plasmid 121718 Plasmid 125454 Plasmid 22115	22 (**68.2**) 34 (**68.8**) 26 (**55.6**) 209 (**69.9**)	4629 (stop) 4014 (stop) 4008 (stop) 2568 (stop)	* Methylibium petroleiphilum* PM1 * Roseobacter denitrificans* OCh 114 * Rhodobacter sphaeroides* 2.4.1 * Ralstonia solanacearum* GMI1000	7e−03 (100) 7e−03 (91) 7e−03 (96) 2e−40 (85)
* Helicobacter pametensis * NCTC1288 (**40.1**)	Plasmid 135582 Virus 97910 Virus 107523 Prophage 170904	66 (**50.0**) 24 (**79.2**) 24 (**79.2**) 26 (**80.8**)	3986 (stop) 2798 (start) 2798 (start) 2800 (start)	* Shewanella baltica* OS155 * Mycobacterium smegmatis * * Mycobacterium smegmatis * * Delftia acidovorans* SPH-1	7e−03 (83) 5e−04 (100) 5e−04 (100) 7e−03 (96)
* Mumia flava * MUSC20	nd	–	–	–	–
* Mycobacterium tuberculosis * 2926STDY5723586 (**65.6**)	Plasmid 27881 Plasmid 90091 Plasmid 17614 Plasmid 21985	38 (**76.3**) 23 (**73.9**) 38 (**81.6**) 22 (**86.4**)	4339 (stop) 4339 (stop) 4335 (stop) 4973 (stop)	* Sinorhizobium meliloti* 1021 * Sinorhizobium medicae* WSM419 * Mesorhizobium loti* MAFF303099 * Ralstonia solanacearum* GMI1000	3e−05 (92) 2e−03 (100) 7e−03 (89) 7e−03 (100)
* Mycobacterium abscessus* subsp* . abscessus* str. 226 (**64.1**)	Plasmid 85650 Plasmid 81573	35 (**82.9**) 22 (**81.8**)	3417 (stop) 320 (stop)	* Rhodobacter sphaeroides* ATCC 17025 * Acidiphilium cryptum* JF-5	8e−06 (94) 6e−03 (100)
* Streptomyces cavourensis * YBQ59 (**72.1**)	Plasmid 81769 Plasmid 20836 Plasmid 127917	26 (**69.2**) 28 (**75.0**) 27 (**81.5**)	3784 (start) 825 (start) 1258 (start)	* Sinorhizobium medicae* WSM419 * Pseudomonas* sp. ADP * Mycobacterium ulcerans* Agy99	7e−03 (96) 5e−04 (96) 2e−03 (96)
* Chryseobacterium* sp. 18 061	nd	–	–	–	–
* Aquificacea *e bacterium isolate MAG 28 Ga0226836_1000157 (**49.4**)	Virus 549 Prophage 171468 Plasmid 14110 Plasmid 14117 Virus 2710	26 (**42.3**) 26 (**42.3**) 43 (**39.5**) 60 (**36.7**) 22 (**13.6**)	2646 (stop) 2646 (stop) 2549 (start) 2296 (start) 1976 (start)	* Bacillus subtilis * * Bacillus subtilis * *Schizaphis graminum* *Diuraphis noxia* * Streptococcus thermophilus *	7e−03 (96) 7e−03 (96) 7e−06 (90) 5e−04 (85) 7e−03 (100)

The putative MGEs were analysed further in terms of their G+C contents, in comparison with those of the genomes of the species in which they were identified. These results, also presented in Table 3, reveal marked differences, in some cases as much as 2.8-fold (plasmid 15088 in *Campylobacter jejuni* NCTC12850) in the G+C content of the putative MGE and that of the genome in which it resides. The longest of the putative MGEs, from plasmid 22115 in '*Empedobacter haloabium'*, has a G+C content that is 2.1 times higher than that of the organism’s genome (Table 3). These results do not, of course, demonstrate the direct participation of these sequences in the HGT of the *pcnB* genes to the NPPC species, but the presence of these putative MGEs is consistent with and supportive of the hypothesis presented here. Taken together, the data presented in the preceding sections argue strongly for the acquisition of *pcnB* genes in the 15 species listed in Table 1 via HGT from species of β- and γ-* Proteobacteria *.

### Are the *pcnB* genes expressed in the NPPC species?

The expression and function of the *pcnB* gene in *E. coli* have been studied in considerable detail both *in vivo* and *in vitro* [12, 50, 51]. Moreover, it was shown recently that the *pcnB* gene is expressed in the δ-proteobacterium, *Geobacter sulfurreducens *, and the PAP activity of the encoded protein was verified [20]. Is there evidence for the expression *in vivo* of any of the *pcnB* genes identified in the present study?

To answer this question, the NCBI Gene Expression Omnibus database (GEO; www.ncbi.nlm.nih.gov/geo) was scrutinized to determine whether transcriptomic or proteomic studies of any of the organisms of interest had been conducted and whether *pcnB* transcripts or the PAP I products thereof were identified in those studies. Transcriptomic or proteomic studies of representatives of several of the NPPC genera, viz. *Campylobacter jejuni *, *Streptococcus pneumoniae *, *Mesorhizobium* sp., *Mycobacterium tuberculosis *, *Mycobacterium abscessus* and *Listeria monocytogenes* were described in the GEO database. However, in none of those studies was the expression of *pcnB* observed.

Transcriptomic studies of two other bacterial species, not listed in Table 1, did indicate the expression of the *pcnB* gene. In *Chlamydia pneumoniae* CWL029, the transcript levels of a gene annotated as *pcnB* were found to vary under different growth conditions [52]. The protein encoded by that gene did contain the PAP I signature sequence, LVGKRFRLAHIRF. Similarly, transcriptome analysis of the spirochaete, *Leptospira interrogans* serovar Copenhageni, identified transcripts of a gene annotated as *pcnB*, whose amounts also varied under different growth conditions [53]. That gene also encoded a protein bearing the PAP I signature sequence, IIGRFFVIHVHIL. Thus, the *pcnB* gene is transcribed in species other than the β-, γ-, and δ-* Proteobacteria *. It should be noted that there is no evidence at this point that either *the Chlamydia pneumoniae* or *Leptospira interrogans pcnB* genes arose by HGT. These results, those described in the preceding sections of this study and the analysis of Martin and Keller [31] strongly suggest that RNA 3′-polyadenylation is widely distributed in the domain Bacteria.

### Evolution of the bacterial NTSFs

**Fig. 4.**
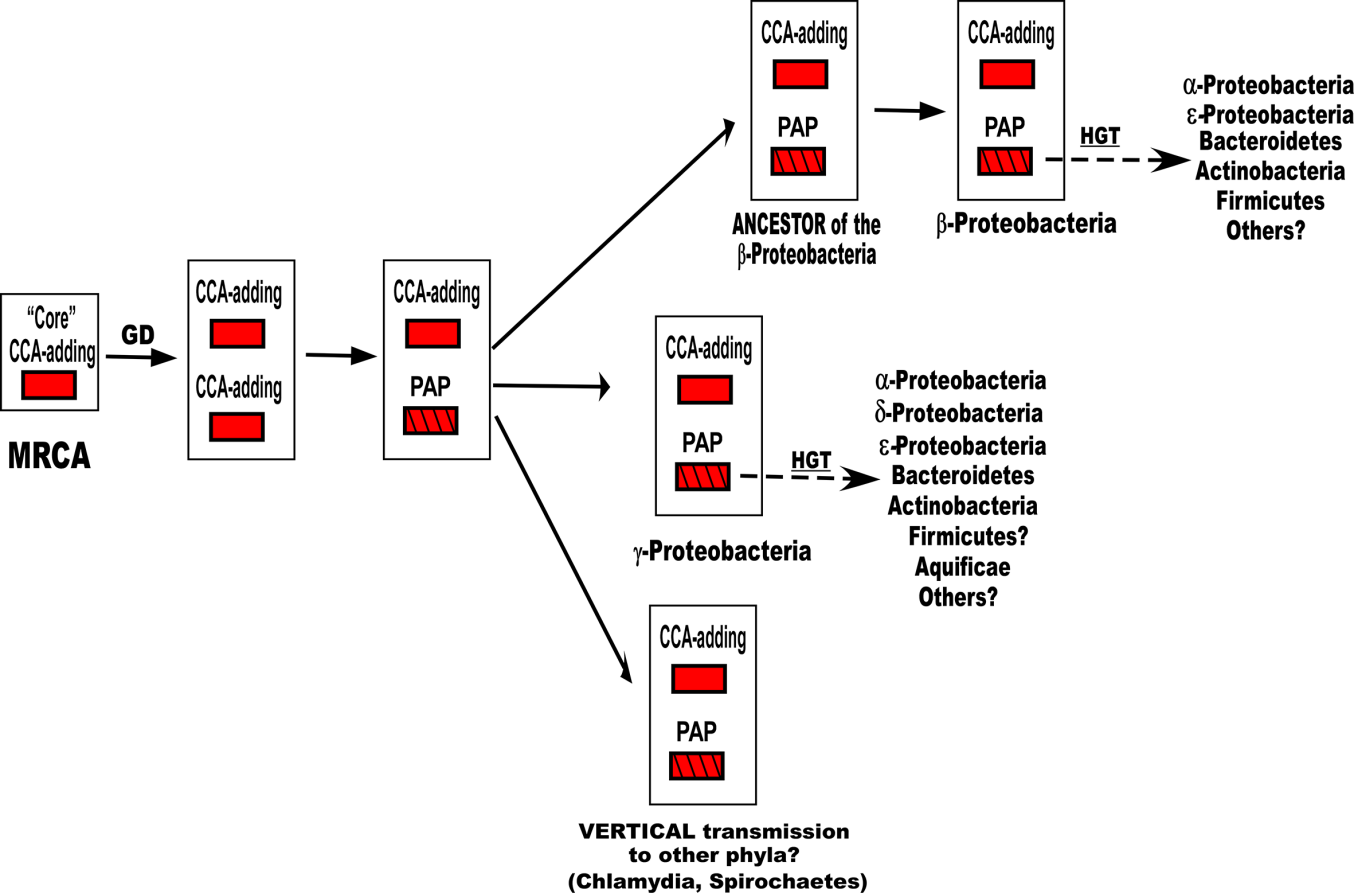
Scheme for the evolution of the bacterial PAPs. The scheme assumes that a core CCA-adding TNT was the ancestor to the modern PAPs. See text for additional details. Although the figure shows schematic representations of the PAP I and TNT proteins, it is the *pcnB* and *cca* genes that would be transferred to recipient species. GD, Gene duplication.

In a previous study, a scheme was proposed for the evolution of the bacterial NTSFs from a common ancestor containing a CCA-adding TNT [1]. That scheme is expanded by the results presented here showing that the PAPs are likely to be present and active in bacterial classes and phyla other than the β, γ-* Proteobacteria *, and that at least some of those PAPs are likely to have been acquired by HGT. A scheme for the evolution of PAPs is shown in Fig. 4.

The scheme posits a most recent common ancestor (MRCA) that contained a *cca* gene and its product, a CCA-adding TNT that lacked the Nrn and CBS domains (core CCA), but no PAP. The MRCA then gave rise to an intermediate species in which a duplication of the *cca* gene occurred. Mutations in one of those two genes subsequently produced *pcnB*. The structural changes required to convert a CCA-adding enzyme to a PAP have been described by the Tomita group in their crystallographic analysis of *E. coli* PAP I [32]. That ancestor then gave rise to the γ-proteobacterial PAPs and later, through an intermediate ancestor, to the β-proteobacterial PAPs. The scheme then posits that the PAPs in the δ-* Proteobacteria* and the NPPC species arose by HGT. The scheme further posits that *pcnB* genes in other phyla (* Chlamydia *, *Spirochaetes* and probably others not listed) arose by vertical transmission, although the horizontal acquisition of *pcnB* by members of some of those phyla cannot be excluded. However, the best blast hits and the corresponding phylogenetic analysis of the putative PAP I proteins identified in *Chlamydia pneumoniae *, *Leptospira interrogans* and several other phyla showed no close relationship to the PAP I proteins from the β- and γ-* Proteobacteria* (data not shown), unlike the situation for the NPPCs.

It is apparent from Fig. 2 that, of the γ-* Proteobacteria* examined in this study, *Xylella fastidiosa* PAP (Xfa in the figure) is closest to the ancestral root of the maximum-likelihood phylogenetic tree. The genus *Xylella* has also been placed near the root of phylogenetic trees based on ribosomal rRNAs, overlapping genes and protein families from the γ-* Proteobacteria* [54, 55]. Thus, it is possible that *Xylella* was among the earliest bacterial genera in which duplication of the TNT gene (*cca*) and its mutation to *pcnB* occurred, and that the *pcnB* gene in that genus is ancestral to the corresponding genes in modern γ-* Proteobacteria *.

**Fig. 5.**
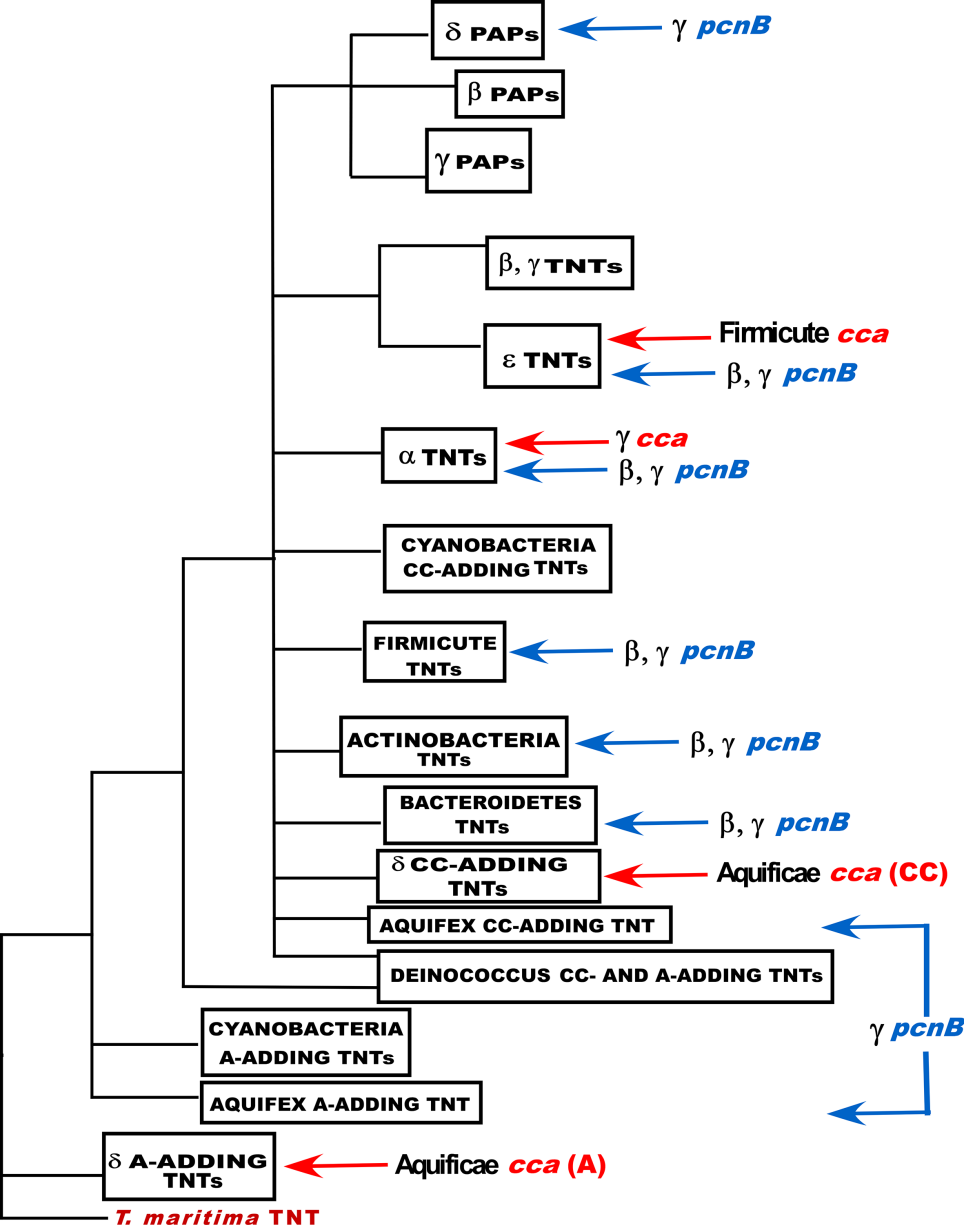
Schematic diagram showing the vertical and horizontal relationships between the TNTs and PAPs discussed in this study [see Table S1 and a previously published paper [1] (Table S1) for the list of species whose proteins were used to construct the figure]. Note that TNTs from six species of *Bacteroidetes* (Table S3) were used to produce this figure, in addition to the sequences mentioned above. A red arrow indicates the horizontal transfer of *cca* (TNT) genes from a member of the phylum or class to the right of the arrow to a member of the phylum or class on the left. A blue arrow indicates the horizontal transfer of *pcnB* (PAP I) genes from a member of the phylum or class to the right of the arrow to a member of the phylum or class on the left. The enzymes listed as TNTs are assumed to be CCA-adding enzymes, except as noted in the figure. As noted in the text, the biological activities of several of the enzymes have been verified experimentally. The sizes of the boxes have no biological significance.

The results obtained in the present study and those presented in a previous paper [1] are summarized in the schematic diagram depicted in Fig. 5, which shows the vertical and horizontal relationships between the TNTs and PAPs analysed in the two studies. This schematic figure is based on the maximum-likelihood phylogenetic tree (Fig. S4), reconstructed from 17 PAP I sequences and 103 TNT sequences, as described in Methods. It is apparent that the *cca* (TNT) genes in species from several phyla (the α- δ- and ε-* Proteobacteria *) were acquired via horizontal transfer from other species [1] and, as documented above, various species of β, γ-* Proteobacteria* were donors for the horizontal acquisition of *pcnB* genes to other bacterial phyla.

As indicated above, this report is not the first demonstration of the presence of PAP I in bacterial taxa other than the β, γ-* Proteobacteria *. In their seminal study, Martin and Keller identified species from a number of bacterial genera that contained proteins bearing the signature sequence [31]. It should be noted, though, that Martin and Keller suggested a different evolutionary origin for the bacterial PAPs. They argued that *pcnB* genes in a variety of bacterial phyla were inherited by vertical transmission and subsequently lost by deletion of the relevant genes [31]. While vertical transmission of *pcnB* genes between phyla other than the *Proteobacteria* cannot be eliminated (see above), the data presented here argue strongly for horizontal transmission to some species.

## Supplementary Data

Supplementary material 1
