# Supplementary material for: Acquisition of pcnB [poly(A) polymerase I] genes via horizontal transfer from the β, γ-Proteobacteria
Source: Microb Genom. 2021 Jan 27;7(2):000508. doi: 10.1099/mgen.0.000508 (PMC8208693; doi:10.1099/mgen.0.000508)
Supplement: Supplementary material 1 [file mgen-7-508-s001.pdf]

TABLE S1 - List of species whose NTSFs are discussed in the text

"Abbrev." refers to the abbreviation used for the species in the figures and tables. "No. of NTSFs" indicates that the genomes of the indicated organisms contain a putative tRNA nucleotidyltransferase and a poly(A) polymerase. **"**Annotated As" refers to the identity of each enzyme as assigned in the NCBI microbial genomes database. The numbers beneath the PAP Protein IDs indicate the positions of the PAP signature sequence in the amino acid sequence of the protein. Only in the case of *Escherichia coli* have the activities of the two NTSFs been verified biochemically. It should be noted, though, that the activities of TNTs from sister organisms to several of those indicated in the table (*Streptomyces coelicolor* [*Mumia flavia* and *Streptomyces cavourensis*] and *Aquifex aeolicus* [*Aquificae* bacterium]) have been demonstrated biochemically (See references in the text).

SPECIES	ABBREV.	NO. OF NTSFs	ANNOTATED AS	PROTEIN ID	LENGTH (aa)
Escherichia coli	Eco	2	PAP<br>TNT	WP_010723084.1 (PAP)<br>(108-120)<br>WP_000708487.1(TNT)	412<br><br>472
Campylobacter jejuni NCTC12850 (ε)	Cje	2	PAP<br><br>TNT	VTQ53567.1<br>(210-221)<br>WP_002868507.1	578<br><br>372
Mesorhizobium sp. (α) isolate N.Ca.ET.004.03.1	Mesp	2	PAP<br><br>TNT	TIX47756.1<br>(110-122)<br>RWM91300.1	474<br><br>422
Rhodobacteraceae bacterium CH30 (α)	Rba	2	PAP<br><br>TNT	WP_124736002.1<br>(95-107)<br>MAS04565.1	446<br><br>420
Pedobacter himalayensis HHS22 (Bacteroidetes)	Phi	2	PAP<br><br>TNT	OOK65942.1<br>(101-113)<br>OOK72508.1	465<br><br>413
Streptococcus pneumoniae NCTC7978 (Firmicute)	Spn	2	CCA-pyro-phosphorylase<br>TNT	VTQ30569.1<br>(90-102)<br>WP_045001916.1	454<br><br>400
Streptococcus dysgalactiae subsp. equisimilis NCTC11565 (Firmicute)	Sdy	2	TNT<br><br>TNT	VTS66379.1<br>(95-107)<br>SQF66810.1	467<br><br>402
Listeria monocytogenes str. 104657	Lmo	2	PAP<br><br>TNT	EAE5926241.1<br>(109-121)<br>EAC7660737.1	472<br><br>345

(Firmicute)					
Empedobacter haloabium (Bacteroidetes)	Eha	2	PAP<br><br>TNT	KAA0215700.1 (95-107)<br>WP_147953035.1	473<br><br>426
Helicobacter pametensis NCTC12888 (ε)	Hpa	2	PAP<br><br>TNT	WP_125080168.1 (95-107)<br>WP_067530290.1	473<br><br>413
Mumia flava MUSC201 (Actinobacteria)	Mfl	2	PAP<br><br>TNT	KHL05611.1 (127-139)<br>WP_039363285.1	521<br><br>482
Mycobacterium tuberculosis str. 2926STDY5723586 (Actinobacteria)	Mtu	2	PAP<br><br>TNT	SGC70168.1 (81-93)<br>WP_003400120.1	451<br><br>480
Mycobacterium abscessus subsp. abscessus str. 226 (Actinobacteria)	Mab	2	PAP<br><br>TNT	SHQ91724.1 (93-105)<br>SHS34529.1	451<br><br>399
Streptomyces cavourensis YBQ59 (Actinobacteria)	Sca	2	PAP<br><br>TNT	RBL87310.1 (93-105)<br>WP_119825607.1	438<br><br>480
Chryseobacterium sp. 18061 (Bacteroidetes)	Chr	2	PAP<br><br>TNT	WP_159761266.1 (101-113)<br>WP_159759966.1	465<br><br>412
Aquificaceae bacterium isolate MAG 28 Ga0226836_10001573 (Aquificae)	Aqb	2	PAP<br><br>PAP<br><br>PAP	RTZ67701.1 (PAP) (76-88)<br>WP_010881416 (CC-adding TNT)<br>WP_010880226 (A-adding TNT)	424<br><br>512<br><br>824

Table S2 – Protein IDs for the polynucleotide phosphorylases (PNPases) used to construct the phylogenetic tree depicted in Figure S3. Abbreviations of species names are as indicated in Table S1 and reference 20.

SPECIES	ID	SPECIES	ID
Lmu	WP_022952822.1	Chr	KFF26834.1
Pae	MXH38198.1	Phi	KIO74643.1
Nba	MXR37111.1	Mesp	TIS56018.1
Ecl	WP_063146521.1	Cje	WP_002882182.1
Cis	WP_159772184.1	Mtu	WP_003414124.1
Eco	AAC76198.2	Aqb	RLD95994.1
Ras	P_113877112.1	Sdy	KKC17316.1
Ype	WP_045123561.1	Rba	MBE2259066.1
Mmo	WP_153641749.1	Hpa	WP_027326719.1
Hso	WP_075320247.1	Eha	TGN27925.1
Pmu	WP_165544675.1	Mfl	WP_100414335.1
Vch	WP_000462068.1	Sca	WP_053559135.1
Xfa	WP_020851437.1	Mab	WP_005076655.1
Acb	WP_025136928.1	Bbr	WP_033458667.1
Bpe	WP_077069198.1	Lmo	HAO5662823.1
Rpi	AGW89358.1	Gsu	BBA70094.1
Las	ODS97385.1	EPRM	WP_085008563.1
Cvi	WP_176219400.1	Pca	WP_011341289.1
Eik	WP_064084312.1	Dso	WP_053550634.1
Nme	WP_024465125.1	Dky	WP_092349863.1
Spn	WP_001118983.1	Mru	WP_072909271.1
		Tma	WP_004081541.1

Table S3 – Bacteroidetes TNTs used in the construction Figs. 5 and S4

SPECIES	ABBREV.	NO. OF NTSFs	ANNOTATED AS	PROTEIN ID	LENGTH (aa)
Empedobacter brevis	Ebr	1	TNT	VDH16430.1	473
Chryseobacterium sp. F5649	Chr	1	TNT	WP_124801980.1	473
Bacteroidetes bacterium	Bba	1	TNT	WP_124801980.1	473
Pedobacter sp. Hv1	Pesp	1	TNT	WP_055132245.1	468
Flavobacterium sp. B17	Fla	1	TNT	WP_042723114.1	473
Cytophaga sp. FL35	Cyt	1	TNT	WP_187457745.1	472

LEGENDS TO SUPPLEMENTARY FIGURES

Figure S1 – Multiple sequence alignment of the PAP and TNT sequences used in the construction of Figure 2.

Figure S2 – Structure of *E. coli* PAP I highlighting the 27 amino acids (orange) implicated by Betat et al. (2014) in the determination of nucleotide specificity. The structure was determined by Toh et al. (2011) and was downloaded from the Uniprot database with accession number 3AQK.

Figure S3 – Maximum likelihood phylogenetic tree generated from the polynucleotide phosphorylase proteins from the species listed in Table S1. The tree was constructed as described in Methods. Bootstrap scores are shown at the nodes. The tree was rooted with the PNPase sequence from *Thermotoga maritima* as the outgroup.

Figure S4 – Maximum likelihood phylogenetic tree generated from the TNT and PAP sequences listed in Tables S1 (this study and reference 20). A total of 17 PAP I sequences and 103 TNT sequences were used in constructing the figure. The analysis also included six Bacteroidetes TNTs (Table S3 above). Bootstrap scores are shown at the nodes. The tree was rooted with the core TNT sequence from *Thermotoga maritima* as the outgroup.

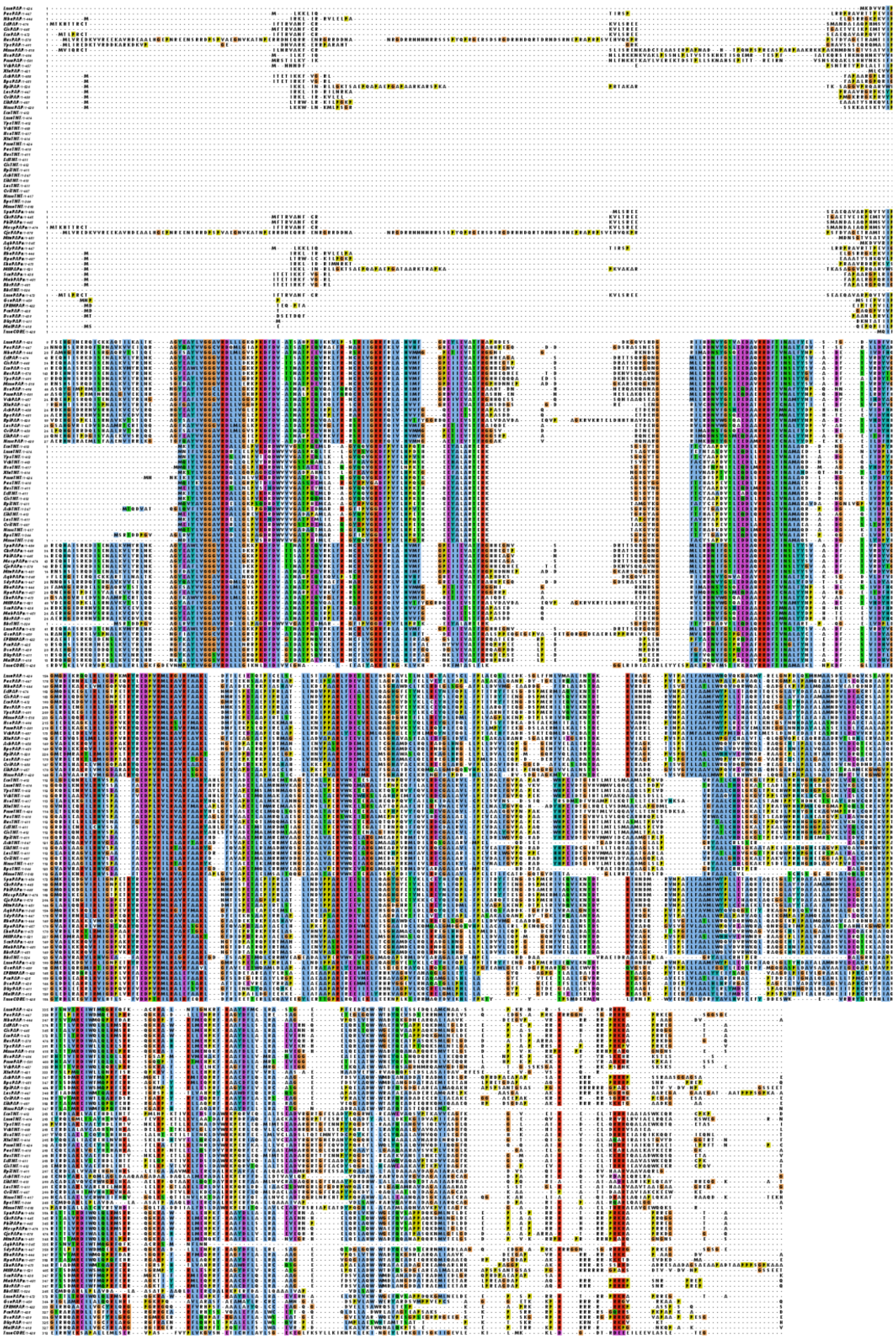

Fig. S1

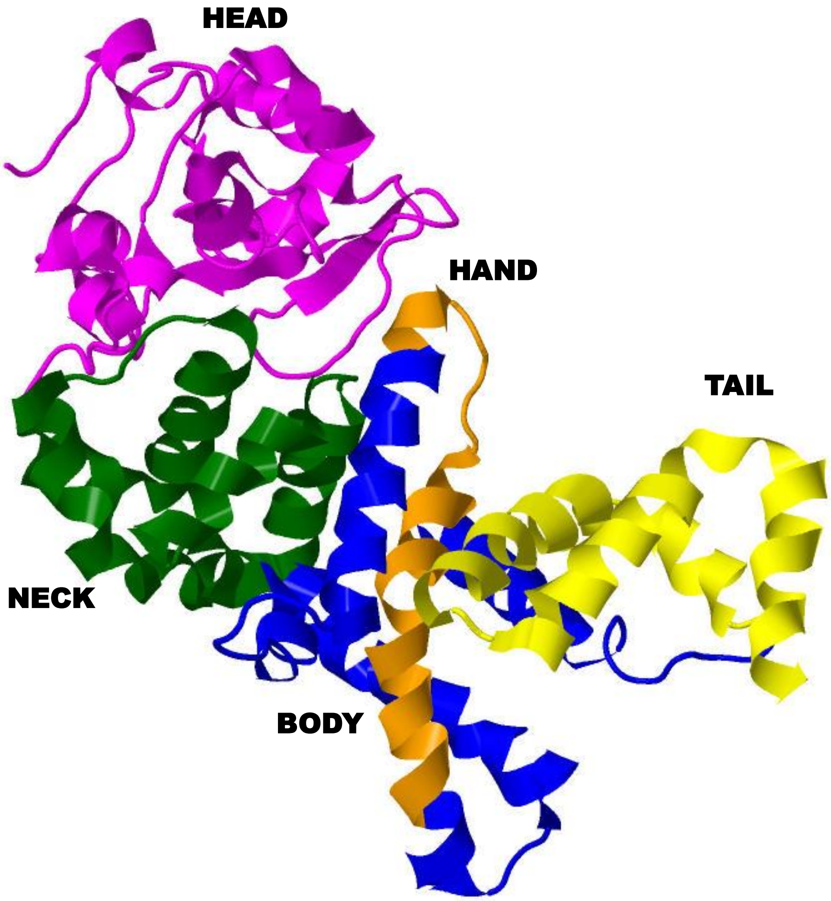

Fig. S2

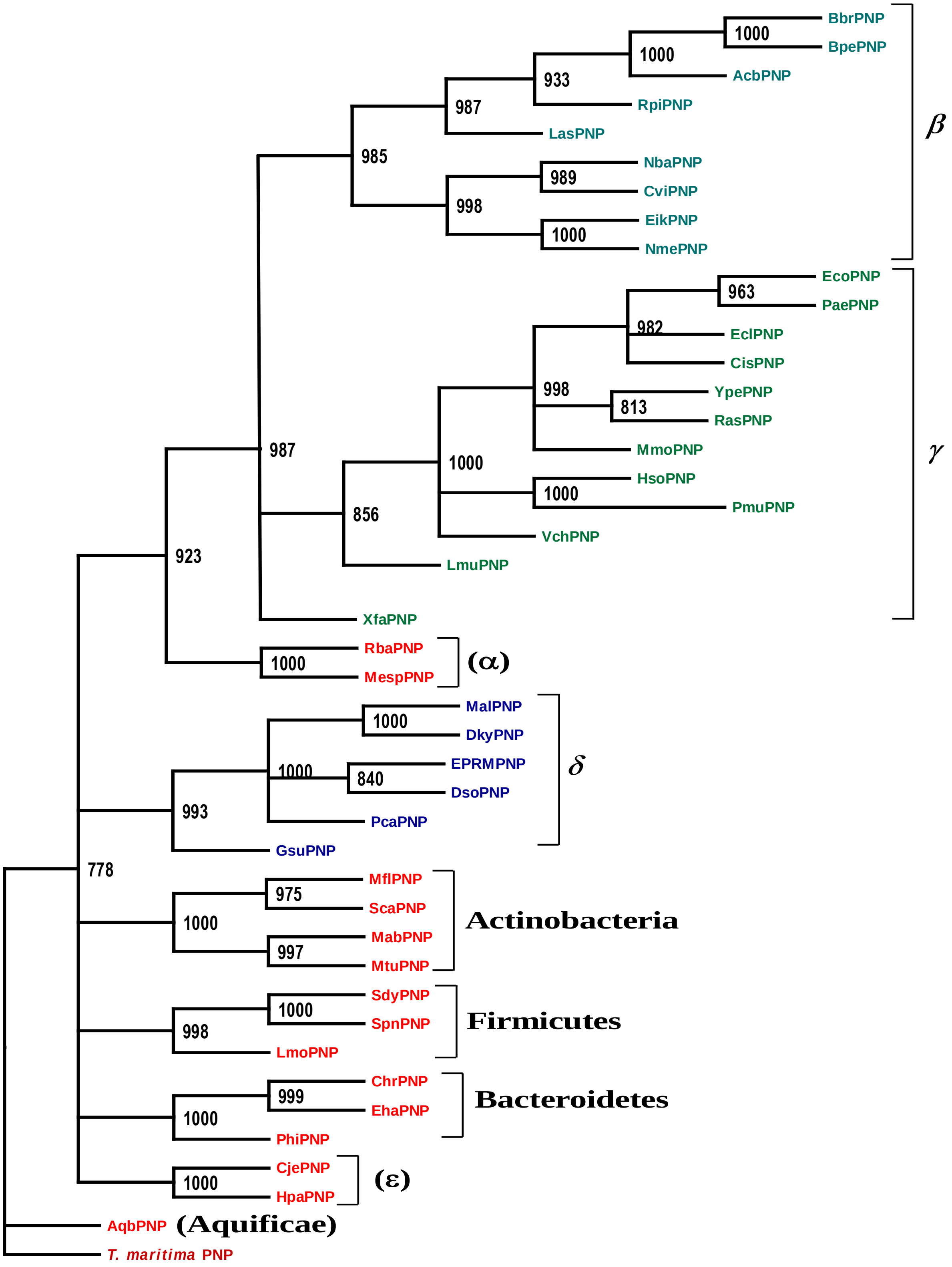
BbrPNP
BpePNP
AcbPNP
RpiPNP
LasPNP
NbaPNP
CviPNP
EikPNP
NmePNP
EcoPNP
PaePNP
EclPNP
CisPNP
YpePNP
RasPNP
MmoPNP
HsoPNP
PmuPNP
VchPNP
LmuPNP
XfaPNP
RbaPNP
MespPNP
MalPNP
DkyPNP
EPRMPNP
DsoPNP
PcaPNP
GsuPNP
MflPNP
ScaPNP
MabPNP
MtuPNP
SdyPNP
SpnPNP
LmoPNP
ChrPNP
EhaPNP
PhiPNP
CjePNP
HpaPNP
AqbPNP
T. maritima PNP
1000
1000
933
987
985
989
998
1000
963
982
998
813
1000
1000
856
987
923
1000
1000
1000
840
993
778
975
1000
997
1000
998
999
1000
1000
β
γ
(α)
δ
Actinobacteria
Firmicutes
Bacteroidetes
(ε)
(Aquificae)

Fig. S3

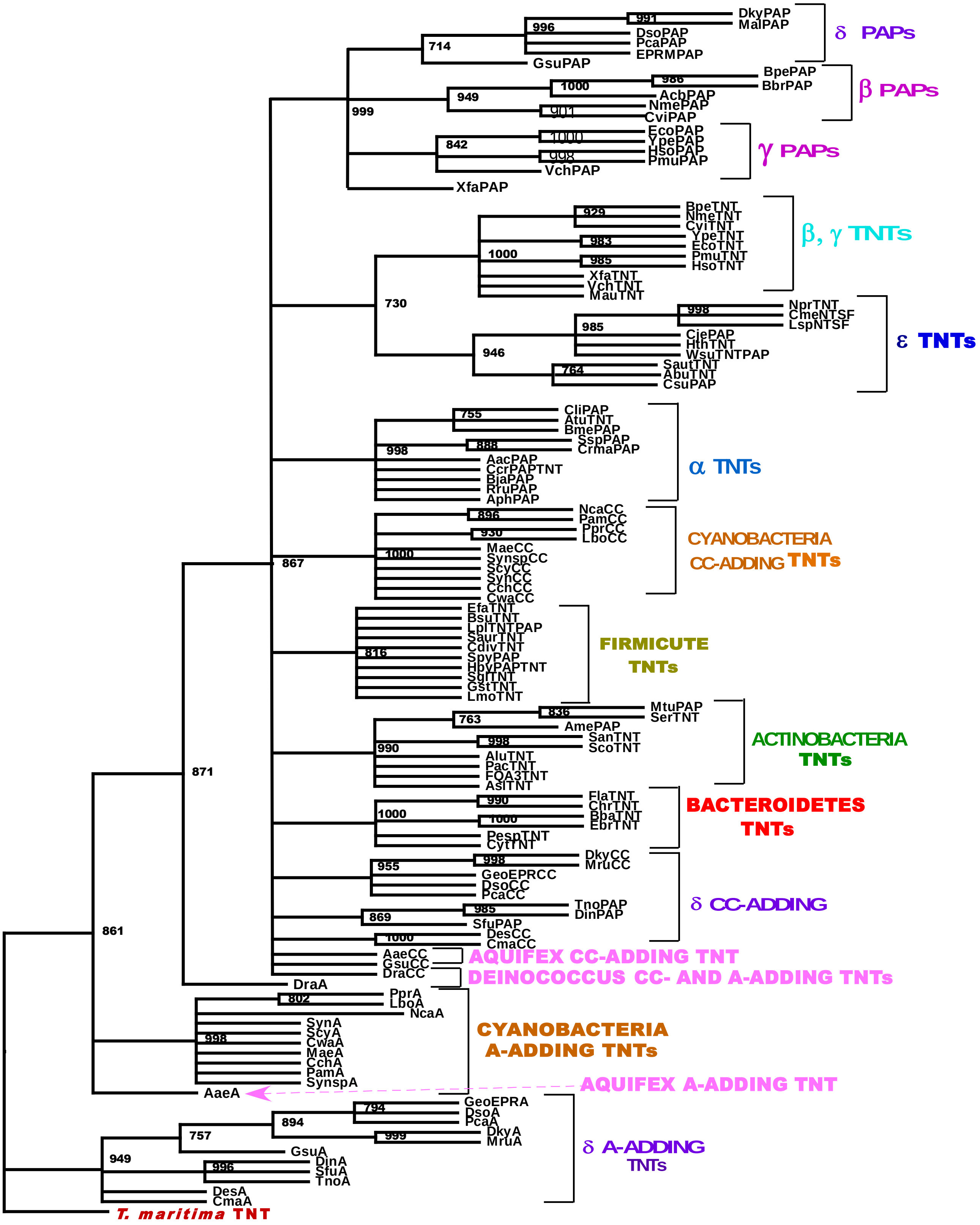

DkyPAP
MalPAP
DsoPAP
PcaPAP
EPRMPAP
GsuPAP
δ PAPs
BpePAP
BbrPAP
AcbPAP
NmePAP
CviPAP
β PAPs
EcoPAP
YpePAP
HsoPAP
PmuPAP
VchPAP
XfaPAP
γ PAPs
BpeTNT
NmeTNT
CviTNT
YpeTNT
EcoTNT
PmuTNT
HsoTNT
XfaTNT
VchTNT
MauTNT
β, γ TNTs
NprTNT
CmeNTSF
LspNTSF
CjePAP
HthTNT
WsuTNTPAP
SautTNT
AbuTNT
CsuPAP
ε TNTs
CliPAP
AtuTNT
BmePAP
SspPAP
CrmaPAP
AacPAP
CcrPAPTNT
BjaPAP
RruPAP
AphPAP
α TNTs
NcaCC
PamCC
PprCC
LboCC
MaeCC
SynspCC
ScyCC
SynCC
CchCC
CwaCC
CYANOBACTERIA
CC-ADDING TNTs
EfaTNT
BsuTNT
LplTNTPAP
SaurTNT
CdivTNT
SpyPAP
HpyPAPTNT
SgrTNT
GstTNT
LmoTNT
FIRMICUTE
TNTs
MtuPAP
SerTNT
AmePAP
SanTNT
ScoTNT
AluTNT
PacTNT
FOA3TNT
AslTNT
ACTINOBACTERIA
TNTs
FlaTNT
ChrTNT
BbaTNT
EbrTNT
PespTNT
CytTNT
BACTEROIDETES
TNTs
DkyCC
MruCC
GeoEPRCC
DsoCC
PcaCC
TnoPAP
DinPAP
SfuPAP
DesCC
CmaCC
δ CC-ADDING
AaeCC
GsuCC
DraCC
AQUIFEX CC-ADDING TNT
DEINOCOCCUS CC- AND A-ADDING TNTs
DraA
PprA
LboA
NcaA
SynA
ScyA
CwaA
MaeA
CchA
PamA
SynspA
CYANOBACTERIA
A-ADDING TNTs
AaeA
AQUIFEX A-ADDING TNT
GeoEPRA
DsoA
PcaA
DkyA
MruA
GsuA
DinA
SfuA
TnoA
DesA
CmaA
δ A-ADDING
TNTs
T. maritima TNT

Fig. S4
